# Secretion of Novel SEL1L Endogenous Variants Is Promoted by ER Stress/UPR via Endosomes and Shed Vesicles in Human Cancer Cells

**DOI:** 10.1371/journal.pone.0017206

**Published:** 2011-02-17

**Authors:** Monica Cattaneo, Lavinia Vittoria Lotti, Simone Martino, Massimo Alessio, Antonio Conti, Angela Bachi, Renato Mariani-Costantini, Ida Biunno

**Affiliations:** 1 Institute for Biomedical Technologies, National Research Council, Milan, Italy; 2 Department of Experimental Medicine and Pathology, “La Sapienza” University, Rome, Italy; 3 Proteome Biochemistry, San Raffaele Scientific Institute, Milan, Italy; 4 Mass Spectrometry, San Raffaele Scientific Institute, Milan, Italy; 5 Department of Oncology and Experimental Medicine, “G. d'Annunzio” University, Chieti, Italy; 6 Aging Research Center (CeSI), “G. d'Annunzio” University Foundation, Chieti, Italy; Vanderbilt University Medical Center, United States of America

## Abstract

We describe here two novel endogenous variants of the human endoplasmic reticulum (ER) cargo receptor SEL1LA, designated p38 and p28. Biochemical and RNA interference studies in tumorigenic and non-tumorigenic cells indicate that p38 and p28 are N-terminal, ER-anchorless and more stable relative to the canonical transmembrane SEL1LA. P38 is expressed and constitutively secreted, with increase after ER stress, in the KMS11 myeloma line and in the breast cancer lines MCF7 and SKBr3, but not in the non-tumorigenic breast epithelial MCF10A line. P28 is detected only in the poorly differentiated SKBr3 cell line, where it is secreted after ER stress. Consistently with the presence of p38 and p28 in culture media, morphological studies of SKBr3 and KMS11 cells detect N-terminal SEL1L immunolabeling in secretory/degradative compartments and extracellularly-released membrane vesicles. Our findings suggest that the two new SEL1L variants are engaged in endosomal trafficking and secretion via vesicles, which could contribute to relieve ER stress in tumorigenic cells. P38 and p28 could therefore be relevant as diagnostic markers and/or therapeutic targets in cancer.

## Introduction

Multiple homeostatic mechanisms that control protein folding and assembly and promote the disposal of defective proteins operate in distinct cellular compartments to afford protection from endogenous proteotoxic stress [1]–[4]. The endoplasmic reticulum (ER) is the folding and assembly site for resident structural proteins and enzymes, as well as for secretory and plasma membrane proteins [5]. This remarkable workload is managed by efficient and high-fidelity protein folding and misfold-correction systems, based on ATP-dependent chaperones and disulfide isomerases, in parallel with quality control mechanisms that allow Golgi transit only to properly folded proteins [6]. Furthermore, clearance of aberrant proteins retained in the ER is mediated through the ER-associated degradation (ERAD) pathway [7], a multi-step process which requires recognition of defective proteins, retro-translocation to the cytosolic side of the ER membrane, ubiquitination and degradation by the 26S proteasome [8].

Nonetheless, the cellular protein-folding capacity and the ERAD pathway may be impaired and/or overloaded by a variety of pathological conditions that perturb energy and calcium homeostasis, increase secretory protein synthesis and/or interfere with protein glycosylation and disulfide bond formation [6], [9]. In such cases the intralumenal accumulation of unfolded/malfolded proteins determines ER stress, which in turn activates a complex cascade of survival signaling pathways, collectively termed unfolded protein response (UPR). This aims at relieving ER stress by attenuating the rate of protein synthesis and by up-regulating the protein folding enzymes, the ERAD machinery and the secretory capacity [6], [10], [11]. If homeostasis cannot be restored, UPR-activated machineries can trigger death/senescence programs [12].

It is increasingly evident that the UPR has a major role in cancer, where it is required to maintain the malignant phenotype and to develop resistance to chemotherapy [13]. In fact cancer cells must adapt to nutrient starvation and hypoxia, which affect cellular redox status and availability of energy from ATP hydrolysis. This is expected to compromise their protein folding capacities, predisposing to ER stress [14]–[16]. Hence, up-regulation of the ERAD-UPR pathways may substantially contribute to the complex cellular adaptations needed for cancer progression [17], [18]. In this regard it is known that many ER-resident proteins are deregulated, post-translationally modified, abnormally secreted and/or cell surface re-localized in various cancer types [13], [19]–[21].

The multifaceted ERAD gene *SEL1L* (sel-1 suppressor of lin-12, *C.elegans*-like) encodes for at least three different protein isoforms, *i.e.*, the canonical ER-resident SEL1LA, a cargo receptor that associates with the E3 ubiquitin-protein ligase HRD1 [22]–[25], and the smaller, recently cloned SEL1LB and -C, that lack the C-terminal SEL1LA membrane-spanning region for insertion into the ER [26]. Several reports have demonstrated that SEL1L protein expression varies in human tumors relative to matched normal tissues, suggesting an involvement in cancer progression [27]–[32].

We report here the identification, characterization and subcellular localizations of two novel anchorless endogenous SEL1L variants, p38 and p28, studied in the breast cancer cell lines SKBr3 and MCF7, the multiple myeloma line KMS11 and the non-tumorigenic lines MCF10A (breast) and 293FT (embryo kidney). We found that: *i.* p38 and p28 are encoded by the 5′ end of the *SEL1L* gene; *ii*. p38 is up-regulated and constitutively secreted in the cancer cells, differently from the non-tumorigenic MCF10A line; *iii.* p28 is expressed only in the poorly differentiated SKBr3 breast cancer line; *iv.* ER stress/UPR strongly enhance p38 secretion in the cancer cells; *v*. N-terminal SEL1L is present in secretory and degradative compartments of SKBr3 and KMS11 cells, and in vesicles released into the extracellular space. Overall, the biochemical and morphological evidence supports the view that SEL1L p38 and p28 are implicated in pathways linking ER stress/UPR to endosomal trafficking and to secretion via extracellularly-shed vesicles. Furthermore the expression of p38 and p28 and their release into the culture medium is upregulated in tumorigenic relatively to non-tumorigenic cells, suggesting cancer-related functions.

## Materials and Methods

### Cell lines, culture conditions, transfections

Human multiple myeloma KMS11, breast cancer MCF7, SKBr3, MDAMB453, lymphoma Namalwa, cervical cancer HeLa and glioblastoma G144, G166, and G179 cells were maintained in RPMI containing 10% fetal bovine serum (Euroclone, Celbio, Pero, Italy). Human embryo 293 FT kidney cells were cultured in DMEM containing 10% fetal bovine serum (Euroclone, Celbio, Pero, Italy). Non-tumorigenic breast MCF10A cells [33] were maintained in MEBM (Lonza, Treviglio, Italy), supplemented with EGF, 20 ng/ml; bFGF, 20 ng/ml; insulin, 10 µg/ml; and hydrocortisone, 0.5 µg/ml. Non-tumorigenic human fetal brain CB660 cells, obtained from Prof. Austin Smith (Cambridge, UK), were maintained in human neural stem cell medium on laminin-coated dishes. Cells were transiently transfected with the indicated plasmids and siRNAs by Lipofectamine 2000 (Invitrogen, S.Giuliano M.se, Italy) and harvested after the indicated time. Cells were treated with DTT (2 mM) for 3 hrs, MG132 (10 µM) for 3 or 22 hrs, cycloheximide (200 µg/ml) for 3, 6, 18 hrs, as indicated.

### Constructs

Tagged TPD52 isoform 1 constructs were generated by cloning the full-length TPD52 isoform 1 coding sequence, fused with a myc or GFP tag at the 3′ end, into pCDNA3.1myc-Hys(-)A or peGFPN3 vectors, respectively (Invitrogen, S. Giuliano M.se, Italy). The Myc-tagged *SEL1LB* constructs were previously described [26].

### RT-PCR

Total RNA was extracted using the TRI Reagent solution (Applied Biosystems, Monza Italy). RNA was reverse-transcribed with SuperScript TM II Reverse Transcriptase (Invitrogen, S. Giuliano M.se, Italy) according to manufacturer's instructions. Semi-quantitative PCR amplifications were performed with 2 µl of RT product per reaction and 0.15 units of Platinum Taq DNA Polymerase High Fidelity (Invitrogen, S. Giuliano M.se, Italy), using a Mastercycler instrument (Eppendorf, Milan, Italy). PCR conditions for all the experiments here described were: denaturation at 95°C for 3 min, followed by 22 cycles at 95°C for 30 seconds, then at 60°C for 72 seconds. The following specific primers were used:

*SEL1LA*: sense: 5′-ctcgctaacaggaggctcagtagtac-3′; antisense: 5′-gccactggcatgcatctgagc-3′

*HRD*1: sense: 5′-ggccagggcaatgttccgc-3′; antisense: 5′-gtccattgcctggagctcc-3′.

*BIP*: sense: 5′-tgcagcaggacatcaagttc-3′; antisense: 5′-cgctggtcaaagtcttctcc-3′

*ATF6*: sense: 5′-ctgatggctgttcaatacac-3′; antisense: 5′-aatgactcagggatggtgct-3′

*CHOP*: sense: 5′-gatggcagctgagtcattgc-3′; antisense: 5′-atgcttggtgcagattcacc-3′

*XBP-1*: sense: 5′-ccttgtagttgagaaccagg-3; antisense: 5′-ggggcttggtatatatgtgg-3′

*GADD45β*: sense: 5′-ggaagagctcgtggcgtgcg-3′; antisense: 5′-gtctcgggcctcggtggtgc-3′

*SEL1LB*: sense: 5′-ccggccccgagaggaggatgcgggtc-3′; antisense: 5′-ggggaaacatagataccatg-3′

*SEL1LC*: sense: 5′-ccggccccgagaggaggatgcgggtc-3′; antisense: 5′-ggggcaatcagccaaactaatca-3′

*HPRT*: sense: 5′-aattatggacaggactgaacgtc-3′ antisense: 5′-cgtggggtccttttcaccagcaag-3′

### Western blotting, immunoprecipitation, analysis of culture supernatants, N-glycosidase F and endoglycosidase H digestion

Monoclonal SEL1L antibody was raised against the N-terminal peptide of human SEL1L [34]. Affinity-purified polyclonal antibody to the SEL1L N-terminus was kindly provided by Dr. H.L. Ploegh [35]. Affinity-purified polyclonal C-terminal SEL1L antibody was raised against a bacterially-expressed recombinant fragment encoding amino acids 575–738 of human SEL1L (Primm, Milan, Italy). Monoclonal anti-vinculin and anti-myc antibodies were purchased from Sigma-Aldrich (Sigma-Aldrich, Milan, Italy). Polyclonal anti-TPD52 was kindly provided by Prof. J. Byrne (University of Sydney, Sydney, NSW, Australia). Cells were lysed in 10 mM Tris-HCl (pH 7.4), 150 mM NaCl, 1% NP40, containing protease inhibitors (Pierce, Celbio, Pero, Italy). Protein concentrations were determined by the Bradford assay; samples were resolved on SDS-polyacrylamide gels, blotted onto PVDF membranes, probed with specific antibodies and developed with ECL (Genespin, Milan, Italy). For immunoprecipitation cell lysates were pre-cleared and incubated with antibodies immobilized on protein G-sepharose (Invitrogen S. Giuliano M.se, Italy). Immunoprecipitates were washed twice with 10 mM Tris-HCl pH 7.4, 150 mM NaCl, 0,25% NP40, once with 5 mM Tris-HCl, and eluted with sample buffer before gel electrophoresis. For analysis of supernatants cells were washed and incubated with OPTIMEM (Invitrogen, S. Giuliano M.se, Italy) for 16 hrs. Supernatants were recovered, centrifuged, precipitated with 10% TCA, re-suspended with 1 M Tris-HCl pH 7.4 and resolved on SDS polyacrylamide gels. All Western blots were performed using the X-BLOT Chamber (www.isenet.it).

For digestion by *N*-glycosidase F (PNGase F) or endoglycosidase H (endo H), cell lysates were denatured by heating at 95°C in 0.05% SDS, 0.1% mercaptoethanol for 10 min and then incubated at 37°C for 1 h with or without PNGase F or endo H (New England Biolabs, Celbio, Pero, Italy), according to the supplier's indications.

### Immunofluorescence microscopy

SKBr3 cells, grown on coverslips, untreated or treated with DTT as described above, were fixed with 4% paraformaldehyde in PBS for 30 min, washed in 0.1 M glycine for 20 min, and permeabilized in 0.1% Triton X-100 for additional 5 min. To investigate the subcellular localizations of SEL1L, the cells were incubated with the N-terminal anti-SEL1L monoclonal antibody [34] and with polyclonal antibodies against the ER marker calreticulin (Affinity Bioreagents, Breda, The Netherlands) and the Golgi marker giantin (Covance, Princeton, NJ, USA). The nuclei were stained with 4,6-diamido-2-phenylindole (DAPI, Sigma-Aldrich, Milan, Italy). Primary antibodies were visualized using fluorescein isothiocyanate-conjugated goat anti-mouse IgG (Cappel Research Products) or Texas-Red-conjugated goat anti-rabbit IgG (Jackson Immunoresearch Laboratories) for 30 min at room temperature. Cells were analyzed using an Apotome Axio Observer Z1 inverted microscope (Zeiss, Oberkochen, Germany), equipped with an AxioCam MRM Rev.3 at 40X magnification. Colocalization of fluorescence signals was analyzed with AxioVision 4.6.3 software. Image analysis was performed using Adobe Photoshop.

### Cryoimmunoelectron microscopy

Cells processed for cryoimmunoelectron microscopy were grown as above and fixed in 2% paraformaldehyde, 0.2% glutaraldehyde in 0.1 M phosphate buffer, pH 7.4, for 2 hrs at 25°C. Cells were scraped off the coverslips, centrifuged, embedded into 10% gelatin (Sigma-Aldrich) in 0.1 M PBS, pH 7.4 and solidified on ice. After infusion in 2.3 M sucrose overnight at 4°C, cell blocks were mounted on aluminum pins and frozen in liquid nitrogen. Ultrathin cryosections (60 nm) were cut at −120°C using an Ultracut EM FC6 cryoultramicrotome (Leica Microsystems, Vienna, Austria), collected with 1% methylcellulose in 1.15 M sucrose and single- or double-immunolabelled with primary antibodies. Bound antibodies were visualized using goat anti-mouse conjugated with 5- or 15-nm colloidal gold (British BioCell International, Cardiff, UK) or by protein-A conjugated with 10-nm colloidal gold (supplied from G.Posthuma and J. Slot, Utrecht, The Netherlands). Immunolabeling was performed with the following primary antibodies: monoclonal anti-SEL1L N-terminus [34], polyclonal anti-SEL1L N-terminus [35], polyclonal anti SEL1L-C terminus, polyclonal anti-calreticulin (Affinity Bioreagents), anti-CD63 monoclonal H5C6, developed by J.T. August and J.E.K. Hildreth, obtained from the Developmental Studies Hybridoma Bank developed under the auspices of the NICHD and maintained by The University of Iowa, Dept. of Biology (Iowa City, IA 52242), and monoclonal anti-c-Myc (Sigma-Aldrich) for *SEL1LBmyc*-transfected 293 FT cells [26]. Single and double immunolabeling were performed as described previously [23], [36]. Cryosections were analyzed with a Philips CM10 transmission electron microscope.

### Off-gel proteins fractionation

Semi-liquid phase isoelectrofocusing fractionation was performed on an Off-Gel 3100 apparatus (Agilent Technologies, Cernusco, IT) using a 24 cm strip with immobilized pH gradient gels in the 4–7 range. Total cell lysate samples (360 µl, corresponding to 1300 µg of proteins) were mixed with 3240 µl of isoelectric focusing buffer (Agilent Technologies) and loaded onto the apparatus. Protein fractionation was performed for 26 hrs with maximum 8000 Volt for a total of 60,000 V/hrs, according to the manufacturer's protocol. The resulting liquid fractions (150 µl) with 0.25 pH range were collected and aliquots of proteins further resolved on 10% acrylamide SDS-PAGE for Western blot probing.

### Protein identification by MALDI-TOF mass spectrometry (MS) analysis

Bands of interest from SDS-PAGE were excised from gels, reduced, alkylated and digested overnight with bovine trypsin (Roche, Milan, Italy), as previously described [37]. One µl (1 µl) aliquots of the supernatant were used for mass analysis using the dried droplet technique and α-cyano-4-hydroxycinnamic acid as matrix. Mass spectra were obtained on a MALDI–TOF Voyager-DE STR mass spectrometer (Applied Biosystem, Foster City, CA). Alternatively, acidic and basic peptide extraction from gel pieces after tryptic digestion was performed and the resulting peptide mixtures subjected to a single desalting/concentration step before MS analysis over Zip-TipC18 (Millipore Corporation, Bedford, MA, USA). Spectra were internally calibrated using trypsin autolysis products and processed via Data Explorer software. Proteins were unambiguously identified by searching a comprehensive non-redundant protein database of the National Center for Biotechnology Information (NCBI, http://www.ncbi.nlm.nih.gov/) and the Mass Spectrometry protein sequence DataBase (MSDB, http://msdn.microsoft.com/en-us/library/ms187112.aspx), selected by default using in house the software programs ProFound v4.10.5 and Mascot v1.9.00, respectively [38], [39]. One missed cleavage per peptide was allowed, and an initial mass tolerance of 50 ppm was used in all searches.

## Results

### Novel ER-anchorless SEL1L variants

**Figure 1:**
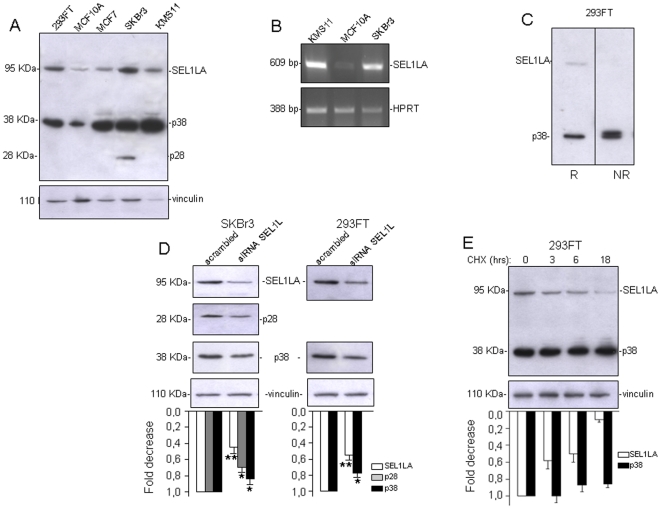
p38 and p28 are related SEL1L variants. A. Western blot analysis: Lysates (50 ug) from different cell lines, including 293FT (embryo kidney), MCF10A (non-tumorigenic breast), MCF7, SKBr3 (breast cancer) and KMS11 (multiple myeloma), were resolved by SDS-PAGE (10%) and probed with monoclonal to SEL1L N-terminus. Vinculin was used as a loading control. In addition to SEL1LA (95 KDa), the N-terminal SEL1L antibody recognized two smaller encoded products, at approximately 38 and 28 KDa (designated p38 and p28). P38 was more abundant than SEL1LA and both were up-modulated in the cancer cell lines relative to MCF10A. P28 was detectable only in SKBr3. Bands above p38, probably corresponding to immature precursors or post-translationally modified products, were occasionally seen in the tested cell lines. The blot is representative of four independent experiments. B. RT-PCR analysis: RNAs from KMS11, MCF10A and SKBr3 cells were analyzed by RT-PCR using primers specific for *SEL1LA*. Signals shown were obtained with 27 cycles for *SEL1LA*. *HPRT* was used as a loading control. *SEL1LA* was up-modulated in the cancer cell lines relative to the non-tumorigenic MCF10A line. The image is representative of three different assays based on independent treatments. C. *Intra/inter*-molecular disulfide bonds analysis of p38. 293FT cell lysates were resolved by SDS-PAGE (10%) under reducing (R) and non-reducing (NR) conditions and blotted with monoclonal anti-SEL1L N-terminus. P38 migrated as a doublet under non-reducing conditions (the lanes comparing p38 migration under reducing and non-reducing conditions are from the same gel). D. Down-modulation of SEL1LA, p28 and p38 by SEL1L small interfering RNA (siRNA): Left panel: SKBr3 cells (3×10^5^) were treated with scrambled siRNA or siRNA specific to SEL1L (siRNA SEL1L) for 48 hrs, followed by a second siRNA treatment for further 48 hrs. Silencing efficiency was verified by Western blot. SEL1LA, p28 and p38 protein levels decreased close to 55%, 30% and 16% respectively compared to cells treated with scrambled siRNA. Vinculin was used as a loading control. Right panel: 293FT cells (6×10^5^) were treated with scrambled siRNA or siRNA-SEL1L for 48 hrs. SEL1LA and p38 protein levels decreased close to 45% and 23% respectively compared to cells treated with scrambled siRNA. Vinculin was used as a loading control. The histograms show values normalized relative to housekeeping signals and expressed as fold modulation relative to controls, densitometric analysis was determined using the Scion imaging program. (www.scioncorp.com). The data are the averages of three independent experiments, ±SD; Student's t-test was used to determine statistical significance *p<0.1; **p<0.05. E. Analysis of p38 and SEL1LA stability: 293 FT cells (6×10^5^) were treated for 3, 6 and 18 hours with cycloheximide (CHX, 200 µg/ml). Aliquots of lysates (50 µg) were resolved by SDS-PAGE (10%) and probed with monoclonal anti-SEL1L and anti-vinculin antibodies. Unlike SEL1LA, which progressively decreased during cycloheximide exposure up to about 90%, p38 levels did not change significantly. The histogram shows the densitometric quantifications obtained through Scion imaging program (www.scioncorp.com). Values were normalized relative to housekeeping signals and expressed as fold modulation relative to untreated samples. The data are averages of two independent experiments, ±SD.

A monoclonal antibody raised against the N-terminus of the SEL1L protein [34] was used to screen a panel of whole lysates from five human cell lines, including MCF7 and SKBr3 (breast cancer), KMS11 (Ig-K-secreting multiple myeloma), 293FT (embryo kidney) and the non-tumorigenic epithelial breast cell line MCF10A. Besides the well-known 95 KDa SEL1LA protein, two additive bands, designated as p38 and p28, were visualized at approximately 38 KDa and 28 KDa (Figure 1A). While p28 was exclusively detected in the SKBr3 line, p38 was strongly expressed, at levels higher than SEL1LA, in all the cancer cell lines tested, with lower levels in MCF10A, where SEL1LA expression (protein and RNA) was also lower (Figure 1A–B). A polyclonal antibody raised against the SEL1L C-terminus, which encompasses the SEL1LA tail anchor for insertion into the ER membrane, detected the 95 KDa SEL1LA protein, but not p38 and p28, although an additional band was evidenced at about 55 KDa, which could indicate the carboxy-terminal fragment resulting from cleavage of the native protein (Figure S1A). Notably the p38 variant was strongly expressed also in other tested cancer cell lines, including Namalwa (lymphoma), MDAMB453 (breast cancer), HeLa (cervical cancer), and G144, G166, G179 (glioblastoma), all of which resulted negative for p28 (Figure S1B). Analysis of SEL1L protein profile in the non-tumorigenic human fetal brain cell line CB660 compared to the three glioblastoma cell lines (Figure S1B) provided further *in vitro* evidence that p38 was indeed expressed at higher levels in tumor cells.

When analyzed in 293FT cells by SDS-PAGE and immunoblot under reducing condition, p38 migrated as a monomer, while it appeared as a doublet under non-reducing conditions (Figure 1C). Thus, while many proteins containing intramolecular disulphide bonds migrate more rapidly under non-reducing conditions due to the more compact native structure [40], p38 migrated faster in reduced than in oxidized state, a phenomenon suggesting that the more slowly migrating form could be engaged in intermolecular disulphide bonds [41]–[43]. The p28 signal appeared as a single band under both reducing and non-reducing conditions (data not shown).

To ascertain that p38 and p28 were *bonafide* endogenous *SEL1L*-encoded proteins, SKBr3 and 293FT cells were transfected with siRNAs targeting the SEL1L N-terminus. The levels of the three SEL1L signals were significantly lower in the cells treated with *SEL1L* versus scrambled siRNAs, with decreases of 55% and 45% for SEL1LA and of 16% and 23% for p38 in SKBr3 and 293FT cells respectively, and of 30% for p28 in SKBr3 (Figure 1D). In 293FT cells, inhibition of protein synthesis by cycloheximide for 3 and 6 hrs, time windows which did not result in cell death, decreased the SEL1LA level by about 50%, but had almost no effect on p38 (Figure 1E). At 18 hrs, cycloheximide caused cell death, concomitant to a drastic depletion of SEL1LA (about 90%), but did not modify the p38 level. This indicates that p38 is more stable than SEL1LA, which may account for the modest p38 depletion by siRNAs to the SEL1L N-terminus.

Altogether, these data indicate that p38 and p28 are SEL1L protein variants encoded by the 5′ end of the *SEL1L* gene. While p38 resulted expressed in all the tested cell lines, with higher levels in the cancer lines, p28 was detected only in the poorly differentiated metastatic breast cancer line SKBr3 [44].

### The p38 and p28 variants display secretory properties

**Figure 2:**
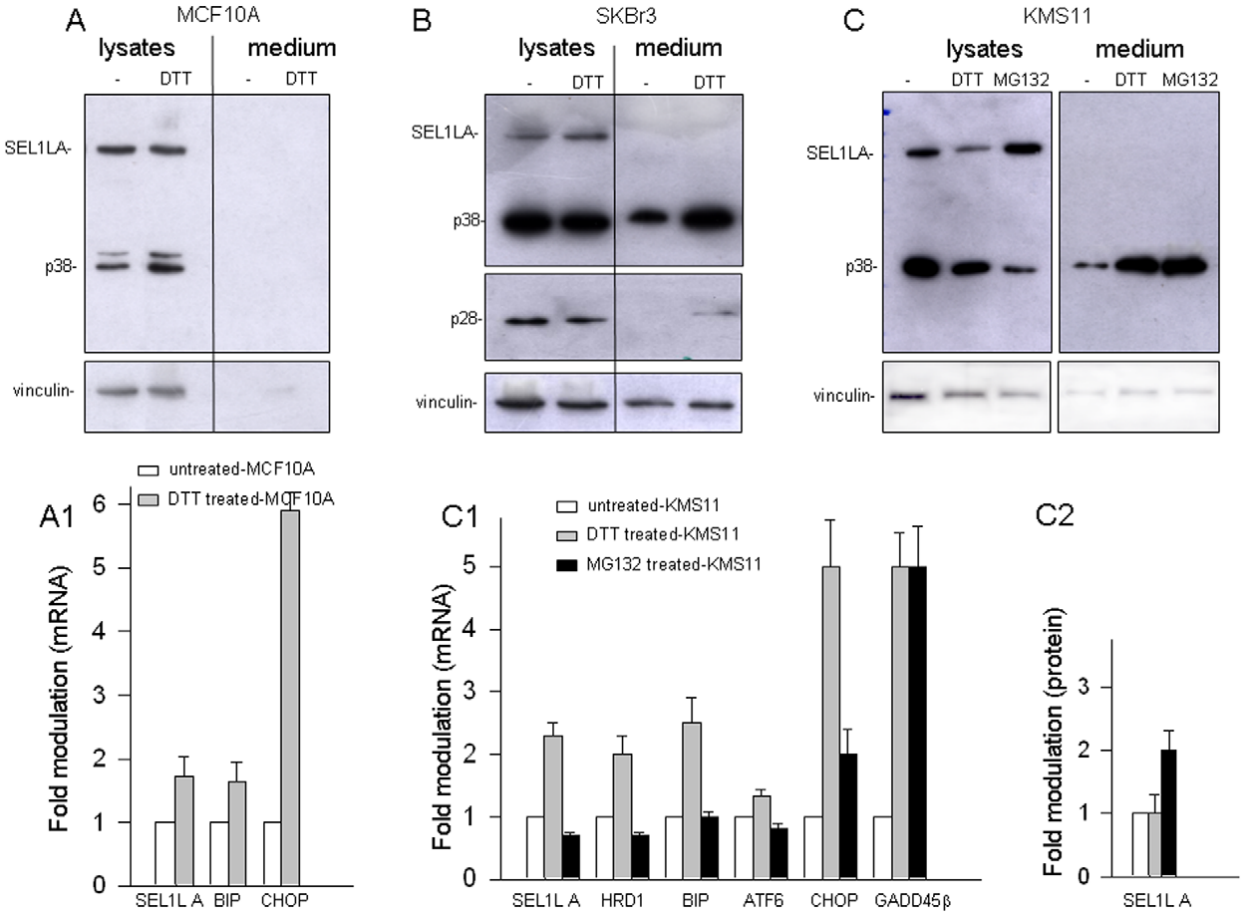
Analysis of p38 and p28 secretion in SKBr3, KMS11 and MCF10A cells exposed to chemical and pharmacological treatments. A. Western blot analysis of untreated and DTT-treated MCF10A cells: MCF10A cells were exposed to DTT (2 mM) for 3 hours and successively maintained for 24 hrs in OPTIMEM. Secreted protein (50 µg) extracted from the culture medium by TCA precipitation, and aliquots of cell lysates (50 µg) were resolved by SDS-PAGE (10%) and blotted with monoclonal anti-SEL1L and anti-vinculin antibodies. P38 was not detectable in the culture medium, both in presence and in absence of DTT. In addition to p38, MCF10A cells showed a higher band, probably corresponding to an immature precursor or post-translationally-modified product. The image is representative of two independent experiments. A1. RT-PCR analysis of untreated and DTT-treated MCF10A cells: RNA was extracted from the samples described in panel A and analyzed by RT-PCR for the UPR response. The histogram shows expression values normalized relative to housekeeping signals and expressed as fold modulation relative to the untreated samples; densitometric analysis was performed by Scion imaging program. UPR activation upon DTT treatment is indicated by up-modulation of *BIP* and *CHOP* and *XBP-1* splicing, concomitantly *SEL1LA* is incremented (gray bar). The corresponding images are shown in Figure S2A. The data are the averages of two different assays based on independent treatments, ±SD. B. Western blot analysis of untreated and DTT-treated SKBr3 cells: Secretion of p38 and p28 was evaluated in untreated and DTT-treated SKBr3 cells. Cells exposed to DTT (2 mM) for 3 hrs or not exposed were maintained for 24 hrs in OPTIMEM. Secreted protein (50 µg) extracted from the culture medium by TCA precipitation, and aliquots of cell lysates (50 µg) were resolved by SDS-PAGE (12%) and blotted with anti-SEL1L and anti-vinculin antibodies. ER stress/UPR strongly promoted secretion of p38 and, to a lesser extent, p28 in the culture medium. The image is representative of five independent experiments. C. Western blot analysis of KMS11 cells treated with DTT and MG132: KMS11 cells were exposed to DTT (2 mM) or MG132 (10 µM) for 3 hrs and successively maintained for 24 hrs in OPTIMEM. Secreted protein (30 µg), extracted from the culture medium by TCA precipitation, and aliquots of cell lysates (50 µg) were resolved by SDS-PAGE (10%) and blotted with anti-SEL1L and anti-vinculin antibodies. Both treatments markedly induced p38 secretion in the culture medium. The image is representative of five independent experiments. C1. RT-PCR analysis of KMS11 cells treated with DTT and MG132: RNA was extracted from the same samples described in panel C and analyzed by RT-PCR for the UPR response. The histogram shows expression values normalized relative to housekeeping signals and expressed as fold modulation relative to untreated samples; densitometric analysis was determined by Scion imaging program. UPR activation upon DTT treatment is indicated by the up-modulation of *ATF6*, *BIP*, and *CHOP* and by *XBP-1* splicing (see Figure S2C), concomitantly the expression of *SEL1LA*, *HRD1* and *GADD45β* is incremented (gray bar). The corresponding images are shown in Figure S2C. MG132 treatment did not trigger UPR activation, as indicated by the absence of *ATF6* and *BIP* un-modulation and lack of *XBP-1* splicing (see Figure S2C), nevertheless, *CHOP* and *GADD45β* were markedly up-modulated and *SEL1LA* and *HRD1* down-modulated (black bars). The data are averages of four different assays based on independent treatments, ±SD. C2. SEL1LA protein expression in KMS11 cells treated with DTT and MG132: The histogram shows SEL1LA protein expression values obtained from the samples described in panel C, normalized relative to housekeeping signals and expressed as fold modulation relative to the untreated sample; densitometric analysis was performed using the Scion imaging program. MG132 determined SEL1LA protein accumulation up to 2 times relative to the control level (black bar). The data are the averages of four independent experiments, ±SD.

To explore the behavior and secretion of p38 and p28 in cells under ER stress/UPR, we analyzed by SDS-PAGE and immunoblot cell lysates and culture supernatants of MCF10A, SKBr3 and KMS11 cells under normal conditions and after treatment with DTT, which causes protein misfolding by reducing disulfide bonds [45] (Figure 2A–C). In all the tested cells DTT triggered ER stress/UPR, assessed by *XBP-1* splicing combined with *BIP*, *ATF6* and *CHOP* up-modulation, and boosted *SEL1LA* mRNA expression (Figure 2A1,C1; Figure S2A–C), while the SEL1LA protein level did not increase significantly (Figure 2A–C, C2). P38, but not SEL1LA, was readily detectable in SKBr3 and KMS11 supernatants, increasing close to three- and five-fold respectively after DTT (Figure 2B–C). No evidence of p38 was detected in MCF10A supernatants, under both normal and DTT-stressed conditions (Figure 2A). P28 was exclusively found after DTT treatment and only in the supernatant of the SKBr3 cell line, which expresses this variant (Figure 2B).

MG132, which determines ER stress through ERAD blockage by proteasome inhibition [46], was tested on the KMS11 myeloma line, being known that the breast cancer cell lines are quite resistant to such treatment [47]. An approximately fivefold increase of p38 in the KMS11 medium occurred after three hours of MG132 exposure, concomitant to a significant increase in *CHOP* and *GADD45β* expression, indicative of ER stress/UPR activation, even if in absence of *XBP-1* splicing and *BIP* and *ATF6* up-modulation (Figure 2C–C1; Figure S2C). MG132 treatment also increased the SEL1LA protein level (Figure 2C, C2), which is consistent with reported evidence that SEL1LA is degraded via proteasome [22]. In contrast, the mRNA levels of *SEL1LA* and of the E3 ubiquitin ligase *HRD1* decreased, suggesting that proteasomal blockage attenuated the transcription of these ERAD genes, whose products associate in the ER membrane-embedded HRD1-SEL1L ubiquitin ligase complex (Figure 2C1; Figure S2C). Prolonged MG132 treatment (22 hours) resulted in cell death, concomitant with up-modulation of *BIP*, *CHOP* and *GADD45β*, indicative of UPR activation (Figure S2C).

Altogether these data indicate that p38 is constitutively secreted in at least two different cancer cell models, *i.e.*, breast cancer and myeloma, and that secretion is up-regulated by ER stress/UPR. This does not occur in the non-tumorigenic MCF10A breast line. Secretion of p28 is restricted to the poorly differentiated breast cancer cell line SKBr3, which expresses this variant, and occurs only after ER stress/UPR.

### Subcellular localizations of SEL1L products

**Figure 3:**
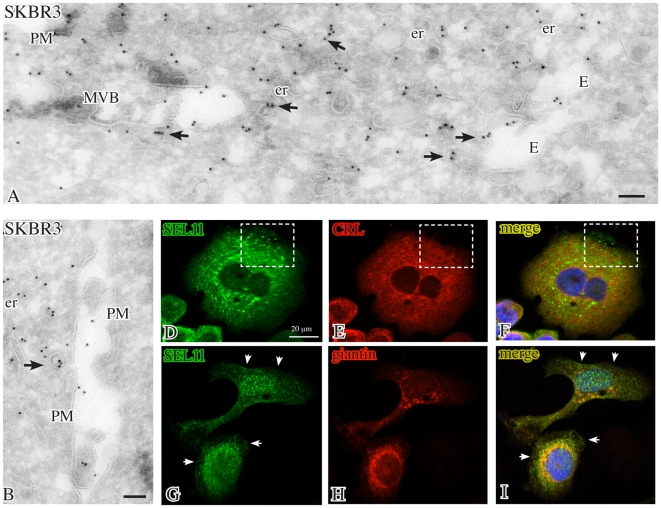
Localizations of SEL1L in untreated SKBr3 cells. Cryoimmunogold electron microscopy of untreated SKBr3 cells labeled with 10 nm gold for N-terminal SEL1L (panels A, B) shows intense staining of vesicles (arrows) dispersed in the peripheral cytoplasm and sometimes associated with multivesicular bodies and endosomes. Immunofluorescence reveals that SEL1L (green) extensively colocalizes (yellow) with the endoplasmic reticulum marker calreticulin and, in a few dots, with the Golgi marker giantin (red), with the exception of discrete cytoplasmic foci (panels D–F, squares) and of the peripheral cytoplasm (panels G–I, arrows). Bars: 0.1 µm; E: endosomes; er: endoplasmic reticulum; MVB: multivesicular body; PM: plasma membrane.

**Figure 4:**
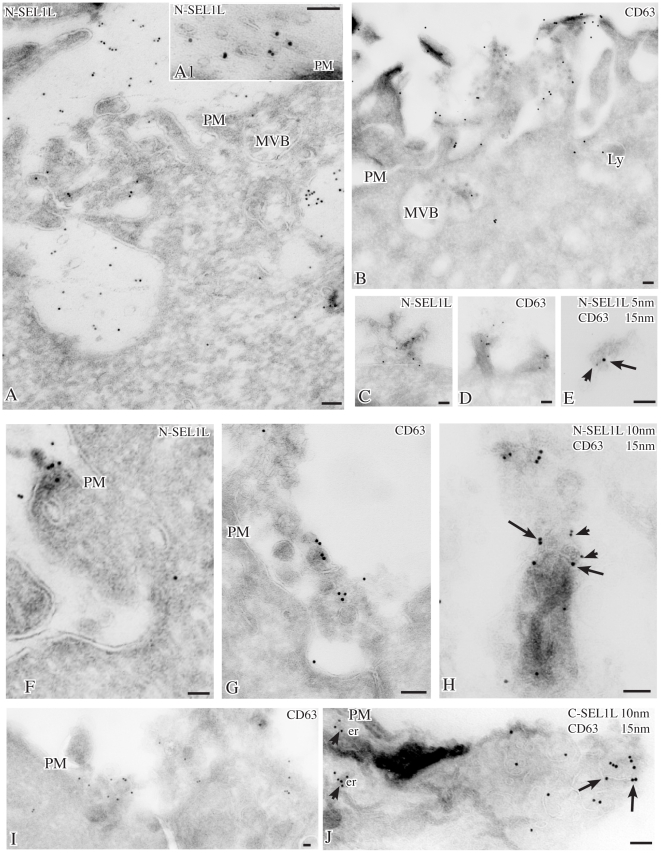
Localizations of SEL1L in DTT-treated SKBr3 cells. After DTT treatment for 3 hrs, gold particles for N-terminal SEL1L (10 nm) are found by cryoimmunogold electron microscopy along the microvilli (panel A) and on membrane-bound vesicles in the extracellular space (panels A, A1) or emerging from the plasma membrane (panels C, F). Similarly, labeling for the tetraspan protein CD63 is detected in multivesicular bodies and in vesicles at the cell surface (panels B, D, G, I). By double immunolabeling (panels E, H), N-terminal SEL1L (5–10 nm gold, arrowheads) localizes in CD63-positive (15 nm gold, arrows) exosomes, while C-terminal SEL1L (panel J, arrowhead) is confined in the endoplasmic reticulum. Bars: 0.1 µm; E: er: endoplasmic reticulum; Ly: lysosomes; MVB: multivesicular body; PM: plasma membrane.

To define the subcellular localizations of the endogenous SEL1L products, SKBr3 and KMS11 cells labeled with the monoclonal or polyclonal antibodies against SEL1L were analyzed by high-resolution immunoelectron microscopy (IEM). Ultrathin cryosections of untreated SKBr3 cells revealed that, in addition to the ER, the monoclonal and polyclonal antibodies against the SEL1L N-terminus [34], [35] labeled peripheral cytoplasmic vesicles, often associated with structures morphologically consistent with late endosomes/multivesicular bodies (MVBs), organelles containing membrane vesicles that can be released extracellularly as exosomes [48] (Figure 3A, B). In agreement, immunofluorescence showed that the immunolabeling obtained with the monoclonal antibody to the SEL1L N-terminus co-localized with the ER marker calreticulin and, in few dots only, with the Golgi marker giantin, but was uniquely present in the peripheral cytoplasm (Figure 3D–I). Double immunolabeling by IEM confirmed that SEL1L codistributed with calreticulin along the ER, while only SEL1L was present in endosomes/MVBs (Figure S3G). In DTT-treated SKBr3 cells the IEM labeling was more evident along the plasma membrane (PM), particularly in association with microvilli, and in 80–200 nm vesicles apparently emerging from the PM and shed extracellularly (Figure 4A–A1, C, F; Figure S3H–I), as observed for the exogenous myc-tagged SEL1LB protein in *SEL1LBmyc*-transfected 293 FT cells (Figure S3J–K) [26].

Immunolabeling with CD63, a marker of lysosomes/MVBs [48], revealed that CD63 and SEL1L were similarly distributed along the microvilli and in membrane vesicles released from the PM (Figure 4D, G, I; Figure S3J), in addition to the localization in lysosomes/MVBs (Figure 4B). Furthermore double immunolabeling revealed that N-terminal SEL1L codistributed with CD63 in some extracellular vesicles (Figure 4E, H; Figure S3 **K**), while the C-terminal SEL1L antibody, which recognizes only the full-length SEL1LA protein, was detected in the ER (Figure 4J). In agreement, immunofluorescence analysis evidenced that N-terminal SEL1L was uniquely localized in peripheral dots and along PM profiles (Figure S3A–F), which were not labeled with the ER marker calreticulin.

**Figure 5:**
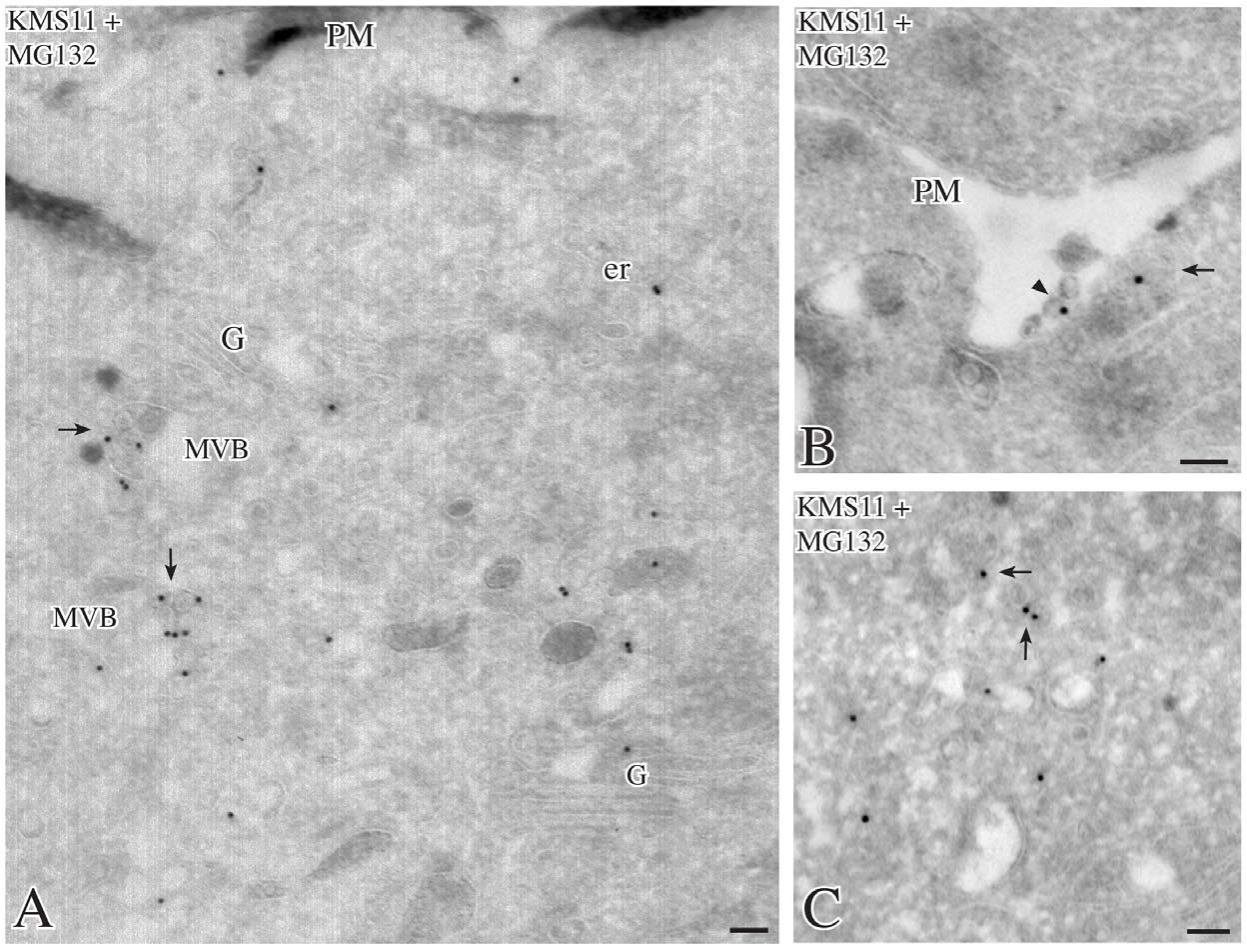
Localizations of SEL1L in MG132-treated KMS11 cells. Cryoimmunogold electron microscopy of MG132-treated cells labeled with 15 nm gold for SEL1L N-terminus shows SEL1L localized mainly in internal vesicles of multivesicular bodies (arrows); with additional labeling in the endoplasmic reticulum, Golgi complex and intracellular vesicles (panels A, C, arrows). In panel B a small SEL1L-labeled multivesicular body (arrow) lies in direct apposition to the plasma membrane. Exosomal-like vesicles, that seem to originate from fusion of this multivesicular body with the plasma membrane, bud into the extracellular space (arrowhead), suggesting that SEL1L is secreted via exosomes derived from the multivesicular body. Bars: 0.1 µm; er: endoplasmic reticulum; G: Golgi apparatus; MVB: multivesicular body; PM: plasma membrane.

Correspondingly, in MG132-treated KMS11 cells, IEM showed that SEL1L was present in vesicles dispersed in the peripheral cytoplasm or associated with late endosomes/MVBs (Figure 5A, C). Furthermore, in areas where MVBs were adjacent to the PM, SEL1L-labeled vesicles appeared to emerge into the extracellular space (Figure 5B).

Overall the morphological data indicate that N-terminal SEL1L localizes in endosomes/MVBs and in vesicles released into the extracellular space, consistently with the SDS-PAGE and immunoblot analysis of the SKBr3 and KMS11 culture supernatants.

### Biochemical characterization of SEL1L variants

We next biochemically characterized the two new SEL1L variants through isoelectrofocusing off-gel fractionation, coupled to Western blot. The isoelectric points (*pIs*) of p38 and p28 ranged between 5.25 and 5.50 (Figure S4A), resulting slightly more acidic than the *pI* of SEL1LA, estimated at 5.8 (http://www.ncbi.nlm.nih.gov/IEB/Research/Acembly). Such *pI* values are compatible with the ultrastructural localization of N-terminal SEL1L labeling in late endosomes/MVBs, which have pH values in the 5 to 6 range [49], [50]. Unlike SEL1LA [23], both p38 and p28 were resistant to N-glycosidase F (PGNase F), which removes all types of N-linked carbohydrates, and to endoglycosidase H (Endo H), which removes high-mannose N-linked oligosaccharides (Figure S4B).

**Figure 6:**
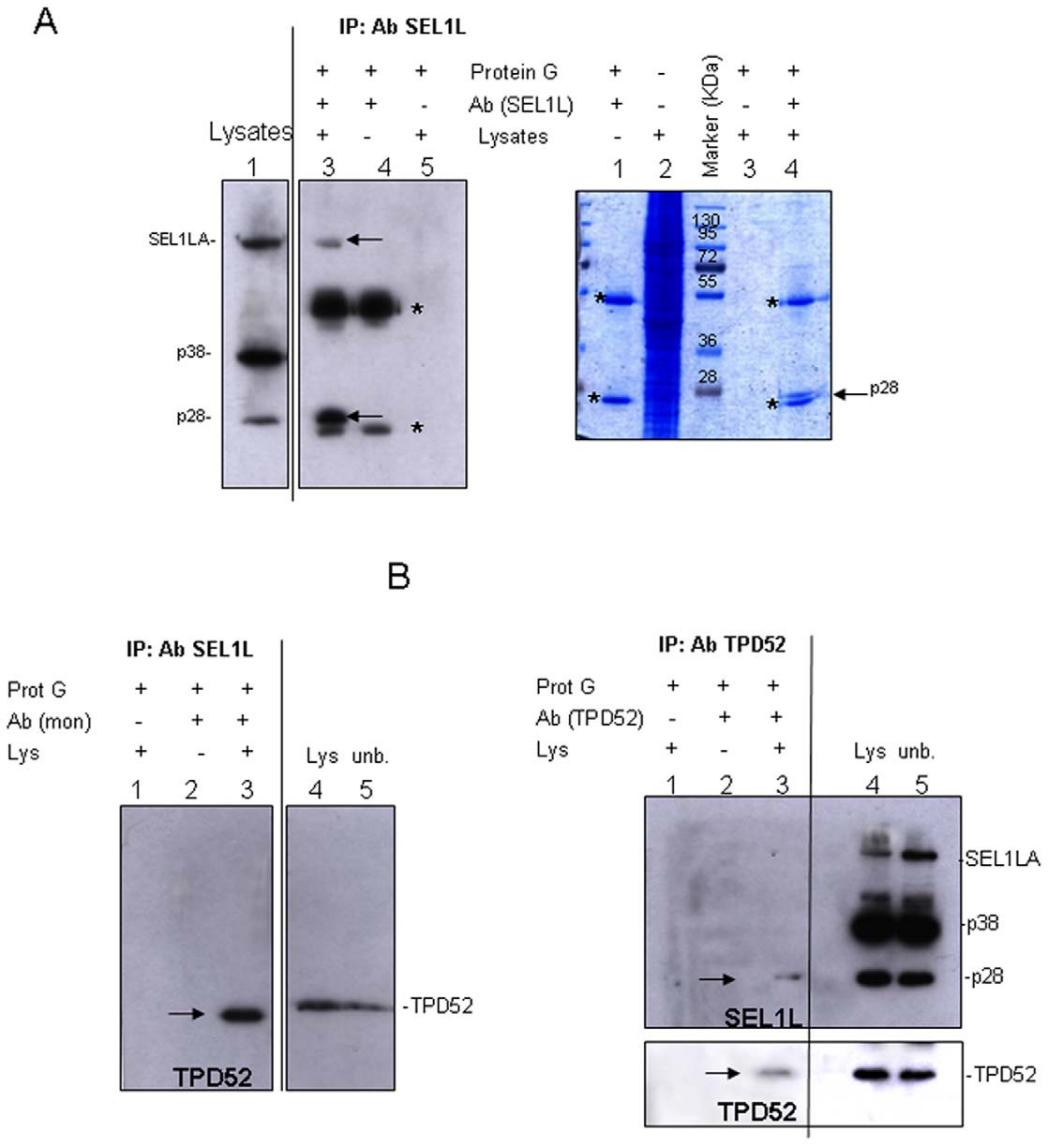
SEL1L and TPD52 immunoprecipitations assays. A. SEL1LA and p28 immunoprecipitation analysis: Left panel: SKBr3 cell lysates (1.4 mg) were immunoprecipitated with monoclonal anti-SEL1L antibody (lane 3), resolved by SDS-PAGE (10%) and probed with monoclonal anti-SEL1L antibody. Lysate aliquots (50 µg, lane 1) were loaded to verify protein expression levels and immunoprecipitation efficiency. Arrows indicate the immunoprecipitated bands, asterisks (*) correspond to heavy and light chains. Absence of signal in controls (lanes 4 and 5) confirms the specificity of the immunoprecipitated bands. Right panel: SKBr3 cell lysates (7.0 mg) were immunoprecipitated with monoclonal anti-SEL1L antibody (lane 4), resolved by SDS-PAGE (10%) and stained with Coomassie brilliant blue. Asterisks (*) correspond to heavy and light chains. The arrow indicates the immunoprecipitated band analyzed by mass spectrometry. Absence of signal in controls (lanes 1 and 3) confirms the specificity of the immunoprecipitated band. B. Analysis of the interaction between SEL1L variants and TPD52: Left panel: SKBr3 cell lysates (1.4 mg) were immunoprecipitated with monoclonal anti-SEL1L antibody (lane 3), resolved by SDS-PAGE (10%) and probed with polyclonal anti-TPD52 antibody. Lysate aliquots (40 µg, lane 4) were loaded to verify protein expression levels and immunoprecipitation efficiency. Lane 5 corresponds to unbound aliquots of the samples loaded in lane 4 (40 µg). The arrow indicates the immunoprecipitated band. Absence of signal in controls (lanes 1 and 2) confirms immunoprecipitation specificity. Right panel: SKBr3 lysates (1.4 mg) were immunoprecipitated with polyclonal anti-TPD52 antibody (lane 3), resolved by SDS-PAGE (10%) and probed with monoclonal anti-SEL1L antibody. Lysate aliquots (40 µg, lane 4) were loaded to verify protein expression levels and immunoprecipitation efficiency. Lane 5 corresponds to unbound aliquots of the samples loaded in lane 3 (40 µg). The membrane was successively re-probed with anti-TPD52 antibody. Arrows indicate the immunoprecipitated bands. Absence of signal in controls (lanes 1 and 2) confirms immunoprecipitation specificity.

To isolate p38 and p28, SKBr3 lysates were immunoprecipitated with monoclonal anti-SEL1L N-terminus, fractionated by SDS-PAGE and either immunoblotted (Figure 6A, left panel) or stained with Coomassie Brillant Blue (Figure 6A, right panel). As shown in Figure 6A, SEL1LA and p28 were immunoprecipitated with different stoichiometric ratios (left panel, lane 3, arrows), but p38, which yielded the most intensely recognized band by immunoblotting, was not recovered in the immunoprecipitates obtained using the same monoclonal antibody. The inability to immunoprecipitate p38 even at small level suggests epitope masking in the native protein, but not in the protein subjected to SDS-PAGE, which could reflect: *i.* protein-protein interactions; *ii.* additive post-translational modifications occurring only in the p38 form; *iii.* nature of the p38 structure. Scaling-up the immunoprecipitations allowed to detect by Coomassie staining a 28 KDa protein band (Figure 6A, right panel, lane 4, arrow), which was subjected to matrix-assisted laser desorption/ionization–time-of-flight mass spectrometry (MALDI-TOF MS) analysis. Surprisingly, MALDI-TOF MS revealed the presence of peptides pertaining to TPD52 (Table 1), a secreted coiled-coil motif-bearing cancer-associated protein implicated in endosomal trafficking and in secretion via membrane-bound vesicles [51]–[61]. TPD52 was readily detectable in SKBr3 cells (Figure S5A), as expected based on the increased gene copy number reported in this cell line [61]. Blast alignment between the coding sequences of *SEL1L* and of three alternatively-spliced *TPD52* isoforms (accession numbers: P55327-1, P55327-2, P55327-3) did not show similarities (data not shown), the monoclonal anti-SEL1L N-terminus did not appear to recognize the myc/GFP-tagged TPD52 isoform 1 (Figure S5B-C) and, in SKBr3 cells, siRNA silencing of *SEL1L* did not affect TPD52 protein level (Figure S5D). ER stress/UPR slightly promoted the release of TPD52 in the SKBr3 culture medium (Figure S5E).

**Table 1:** TPD52 identification by MALDI-TOF MS analysis.

Band	IPI	Acc.No. Swissprot	Entry name	Protein name	Theoretical pI/Mr	Mowse Score	Meas Pept	Match Pep.	Seq. Cov
1	IPI00619958	P55327	Human TPD52	Tumor protein D52	4.79/24327	177	76	18	82%

IPI: International Protein Index; Acc. No.: Accession number; Seq. Cov.: sequence coverage; *pI*: isoelectric point; Mr: relative molecular mass; MOWSE: MOlecular Weight SEarch, probability based score used by the Mascot software which revealed higher probability with TPD52 isoform 1; Meas. Pep.: measured peptides; Matc. Pep.: matching peptides.

To investigate whether SEL1LA and/or p28 physically interacted with TPD52, SKBr3 lysates were immunoprecipitated with either anti-SEL1L N-terminus or anti-TPD52 antibodies and conversely analyzed by Western blot using anti-TPD52 or anti-SEL1L (Figure 6B). TPD52 was immunoprecipitated using monoclonal anti-SEL1L (left panel, lane 3, arrow); reciprocally, in spite of the low immunoprecipitation efficiency, p28, but not SEL1LA, was recovered using anti-TPD52 (right panel, lane 3, arrows). This suggests that in SKBr3 cells p28 and TPD52 interact, with a stoichiometric imbalance that might reflect differences in expression level and/or immunoprecipitation efficiency.

Overall, these results indicate that the *pI*s of p38 and p28 are compatible with their presumed localization in endosomes/MVBs, that both are underglycosylated, and that p28 interacts with the cancer-associated protein TPD52, implicated in endosomal trafficking and secretion via vesicles [51]–[61].

## Discussion

We report here two new anchorless endogenous SEL1L variants, p38 and p28, identified in lysates of different cell lines, including KMS11 (multiple myeloma), 293FT (embryonic kidney), MCF7, SKBr3 (breast cancer) and MCF10A (non-tumorigenic breast). In addition to the signal of the canonical ER-resident SEL1LA protein, we found distinct additive bands at approximately 38 KDa (p38) and 28 KDa (p28). While p28 was detectable only in the poorly differentiated breast cancer line SKBr3, p38 was expressed in all the cell lines tested, at levels higher than SEL1LA and with stronger signals in cancer cells. In this regard, recent studies of SEL1L expression in human colorectal tumors revealed higher p38 levels in adenomas compared to matched normal colonic mucosa, suggesting an association between upregulation of p38 and *in vivo* colonic tumorigenesis (Ashktorab et al., unpublished results).

Recognition by antibodies to the SEL1LA N-terminus, but not to the C-terminus, and RNA interference assays indicate that p38 and p28 are low molecular mass N-terminal SEL1L forms, that could originate either from splicing events at the 5′ end of the *SEL1L* pre-mRNA transcript, as the recently reported *SEL1LB* and *–C* isoforms, cloned from RNA extracted from normal peripheral blood lymphocytes [26], or, more likely, from proteolytic cleavage of the ER-resident SEL1LA. In this regard it is relevant that bioinformatic analysis predicts several cleavage sites in the SEL1LA protein sequence (peptide cutter program, http://expasy.org/tools/peptidecutter). The hypothesis that p38 could originate from SEL1LA cleavage would be consistent with the evidence that DTT treatment upregulates *SEL1LA* mRNA, but not SEL1LA protein level, which could suggest either that DTT, by altering terminal folding, compromises SEL1LA stability, or that most of SEL1LA undergoes cleavage to p38, that is then secreted. In this case the band at about 55 KDa evidenced in SKBr3 cells using antibody to the SEL1L C-terminus could represent the carboxy-terminal fragment obtained after cleavage of p38. Furthermore, we recently observed that miR183 negatively regulates both SEL1LA and p38, a finding supporting the view that at least p38 results from a post-translational modification of the SEL1LA product (Biunno, unpublished results). Thus, while SEL1LB and –C are generated from alternatively-spliced mRNAs expressed at low levels and up-modulated in cancer cells (Figure S6) and under ER stress [26], the two new soluble SEL1L forms, abundantly expressed in cancer cells, could likely originate from proteolytic cleavage of SEL1LA.

As SEL1LB and -C, p38 and p28 lack the C-terminal SEL1LA membrane-spanning region, but are predicted to retain several sel1-like tetratricopeptide repeats, known to serve as protein-protein interaction modules [26], [62]–[64]. Unlike SEL1LA [23], both p38 and p28 are PGNase F and Endo H resistant, which may reflect the lack of the N-linked glycosylation sites at the SEL1LA C-terminus [65], [66], while the N-linked glycan identified in the SEL1LA N-terminus [67] could be proximal to or beyond the splicing or cleavage sites. The lack of asparagine-N-linked high-mannose-type carbohydrate chains implies major differences in the folding, oligomerization, sorting, and transport of p38 and p28 relative to SEL1LA [68]. The modest depletion of the two new forms, especially p38, after RNA interference or blockage of protein synthesis, points to their higher stability compared to SEL1LA.

Most interestingly, p38 is constitutively secreted in the culture media of the SKBr3 and KMS11 cancer cell lines, and secretion is strongly augmented by ER stress or proteasomal blockage. The p28 form is detectable in the SKBr3 culture medium only after ER stress. Importantly, no SEL1L immunoreactive bands are found in the MCF10A culture medium under normal and ER-stressed conditions, suggesting that, at least in cells of breast epithelial origin, secretion of the two soluble SEL1L forms is associated with the tumorigenic phenotype.

Overall, the structural and functional properties of endogenous p38 and p28 resemble those of the previously cloned exogenous SEL1LC and -B in isoelectric point, high stability and localization in endosomes/MVBs and secretory vesicles [26]. As SEL1LB and -C, also p38 and p28 are predicted to be structurally related to secreted bacterial virulence factors involved in pathogen-host interactions, such as the *Legionella pneumophila* LpnE, EnhC and LidL proteins and the *Helicobacter pylori* cysteine-rich protein A (HcpA) [26], [63]. LpnE is implicated in the ability of *L. pneumophila* to establish infection and/or manipulate host cell trafficking events, and its sel-1 like repeats, that interact with proteins containing Ig-like domains, are necessary for host cell invasion [69]. HcpA is a *β*-lactamase with hydrolytic activity, implicated in drug resistance and proinflammatory/immune responses [70]–[72].

Morphological analyses indicate that in SKBr3 and KMS11 cells N-terminal SEL1L immunolabeling is detectable not only in association with the ER, but also in endosomes/MVBs, along the PM profiles and within peripheral cytoplasmic or extracellular vesicles. These diverse subcellular localizations were observed using two distinct antibodies to the SEL1L N-terminus, while an antibody to the SEL1L C-terminus, unique to the ER-resident SEL1LA, confirmed only the immunolabeling of the ER. However, the N-terminal SEL1L antibody cannot discriminate between p38 and p28, and the distribution of the N-terminal SEL1L immunoreactivity in the different subcellular compartments was similar in cell lines that express both p38 and p28, such as SKBr3, or only p38, such as KMS11. By IEM, the N-terminal SEL1L labeling in the vesicles shed by SKBr3 and KMS11 cells appears to increase after induction of ER stress, in agreement with the SDS-PAGE and immunoblot analysis of the culture supernatants. Furthermore, the co-immunoprecipitation data obtained in SKBr3 cells suggest a functional parallelism between p28 and the TPD52 family proteins, cancer markers that localize to endosomes/MVBs and act as regulators of membrane trafficking in exocytic pathways [51]–[61]. MVBs are endosome-derived multivesicular organelles containing hydrolases, which may evolve into lysosomes or into secretory organelles [73], [74]. The localization of the N-terminal SEL1L immunolabeling in endosomes/MVBs is consistent with the slightly acid *pI*s of p38 and p28 [49], [50]. In this regard, it is known that ER proteins that escape ERAD, as well as ERAD components, can be targeted to the endosomal pathway for lysosomal or basal autophagic degradation [74]–[76]. Alternatively, endosomes/MVBs can be involved in exocytosis, which may contribute to relieve ER stress through the expulsion of damaged proteins and membrane constituents [77]–[79]. The extracellularly-released vesicles containing N-terminal SEL1L products appear to be heterogeneous in origin, deriving from vesicles segregated within MVBs and discharged upon fusion with the plasma membrane (exosomes), and from small plasma membrane protrusions shed after fission of the stalk (shedding vesicles) [78]–[80]. Notably, in agreement with our data, a recent report includes SEL1L peptides among the proteins identified by mass spectrometry in purified Rab27b-secretory vesicles of MCF7 breast cancer cells [81]. Interestingly, Rab27b, a GTPase implicated in PM delivery and fusion of different secretory vesicle types, is reported to be present in CD63-containing multivesicular elements located adjacent to the PM and in exosomes [81], [82].

Vesicles participate in plasma membrane traffic and in intercellular communication, enabling the horizontal transfer of membrane and/or cargo molecules, including proteins and mRNAs, from cell to cell or to the extracellular compartment, where they can dissolve, releasing their contents. In this regard shed vesicles provide platforms for integrated multisignaling, required for rapid phenotype adjustments in cell populations [80]. Being extracellularly released, p38 and p28 could be found in abnormal amounts in biological fluids, and could be potentially developed as tumor markers. In this regard, it is intriguing that bayesian network modelling of microarray and mass spectrometry data identified an N-terminal SEL1LA sequence as a putative serum biomarker of prostate cancer [83].

It could be speculated that p38 and p28, which seem to be secreted only in tumorigenic cells, might be involved in hydrolytic and proinflammatory processes associated with cancer-related autocrine/paracrine signalling induced by ER stress. In this respect, signaling via tumor-released vesicles is implicated in processes that facilitate metastasis, such as extracellular matrix remodelling, angiogenesis and migration [80]. Thus, p28 and p38 could represent new tumor markers and provide potential targets for cancer therapy.

## Supporting Information

Figure S1 **P38 and p28 are not identified by antibody against the SEL1L C-terminus and are detected in cancer cells of various origin by antibody against the SEL1L N-terminus.** A. P38 and p28 are not recognized by polyclonal antibody against the SEL1L C-terminus: Lysates (50 µg) from 293FT (embryo kidney) and SKBr3 (breast cancer) cells were resolved by SDS-PAGE (10%) and probed with polyclonal anti-SEL1L C-terminus. Vinculin was used as a loading control. The polyclonal C-terminal SEL1L antibody recognized the ER-resident SEL1LA protein (95 KDa), but not the p38 and p28 forms. The blot is representative of three independent experiments. B. p38, detected with monoclonal antibody against the SEL1L N-terminus, is more evident in cancer cells of various origins relative to a normal human fetal brain cell line: Lysates (50 µg) from Namalwa (lymphoma), MDAMB453 (breast cancer), HeLa (cervical cancer), G144, G166 and G179 (glioblastoma) and CB660 (human fetal brain) cells were resolved by SDS-PAGE (10%) and probed with monoclonal anti-SEL1L N-terminus. Vinculin was used as a loading control. P38 was expressed at much higher levels in the tested cancer cell lines relative to CB660, while p28 was undetectable. A higher band of approximately 60 KDa may represent an additional SEL1L-related form expressed in glioblastoma cell lines.
(TIF)

Figure S2 **UPR studies in MCF10A and SKBr3 cells treated with DTT and in KMS11 cells treated with DTT and MG132.** A. RT-PCR analysis of DTT-treated MCF10A cells: UPR activation was analyzed by RT-PCR in the samples described in Figure 2A1. UPR activation was confirmed by *XBP-1* splicing and up-modulation of *BIP* and *CHOP*. *HPRT* serves as internal control. The image is representative of two different assays based on independent treatments. B. RT-PCR analysis of DTT-treated SKBr3 cells: RNA was extracted from the samples described in Figure 2B and analyzed by RT-PCR for the UPR. UPR activation was confirmed by *XBP-1* splicing and *CHOP* up-modulation, concomitantly with increase of *SEL1LA*. *HPRT* serves as internal control. The image is representative of five different assays based on independent treatments. C. RT-PCR analysis of DTT- and MG132-treated KMS11 cells: UPR activation was assessed by RT-PCR on KMS11 cells treated with DTT or with MG132. UPR activation upon DTT treatment was confirmed by *XBP-1* splicing and *CHOP*, *BIP* and *ATF6* up-modulation; concomitantly *SEL1LA* also increased. MG132 treatment for 3 hrs resulted in an increase of *CHOP* and *GADD45β*, but there was no evidence of *XBP-1* splicing and *BIP* and *ATF6* modulation. Concomitantly, *SEL1LA* and *HRD1* decreased. After 22 hours of MG132 treatment, *BIP*, *CHOP* and *GADD45β* increased. *HPRT* serves as internal control. The image is representative of five different assays based on independent treatments.
(TIF)

Figure S3 **Localizations of SEL1L in DTT-treated SKBr3 cells and of myc-tagged exogenous SEL1LB in transfected 293FT cells.** Immunofluorescence shows that in DTT-treated SKBr3 cells N-terminal SEL1L (green) intensely labels peripheral areas negative for the endoplasmic reticulum marker calreticulin and for the Golgi marker giantin (panels A–F). Cryoimmunogold electron microscopy of DTT-treated SKBr3 cells shows N-terminal SEL1L labeling in multivesicular bodies (panel G, arrowhead), on endoplasmic reticulum profiles, identified by calreticulin (panel G arrows), and in vesicles released from the plasma membrane after fission of the stalk (panels H-I, arrows point to stalks). Similarly, in *SEL1L-Bmyc*-transfected 293FT cells, exogenous myc-tagged SEL1LB labeling was detected along plasma membranes and in vesicles emerging from plasma membrane (panels J–K, arrows). Bars: 0.1 µm; er: endoplasmic reticulum; MVB: multivesicular body; PM: plasma membrane.
(TIF)

Figure S4 **Biochemical characterization of SEL1L variants.** A. Off-gel electrophoresis and Western blot analysis: Off-gel electrophoresis coupled with Western blot analysis was used to analyze the *pIs* of p38 and p28. The proteins extracted from the medium of DTT-treated SKBr3 cells were fractionated according to their *pI* using an Off-gel 3100 fractionator (Agilent Technologies) and aliquots of these fractions were analyzed by Western blot with monoclonal anti-SEL1L antibody. Both p38 and p28 (arrows) were detected in the six^th^ fraction, corresponding to the *pI* range of 5.25–5.50. B. N-glycosidase F (PGNase F) and endoglycosidase H (Endo H) digestions: SKBr3 cell lysates (80 µg) were incubated with endo H (*H*) and PGNase F (*F*), fractionated on SDS-PAGE (10%) and blotted with monoclonal anti-SEL1L antibody. Both p38 and p28 are PNGase F and endo (H) resistant. Note the mobility shifts of treated SEL1LA (see arrow).
(TIF)

Figure S5 **TPD52 analysis.** A. TPD52 protein expression: Lysates (50 µg) from different cell lines, including 293FT (embryonic kidney), KMS11 (multiple myeloma), MCF7, MDAMB453, and SKBr3 (breast cancer) were resolved by SDS-PAGE (10%) and probed with polyclonal anti-TPD52 antibody. Vinculin was used as a loading control. SKBr3 cells over-expressed TPD52. The blot is representative of three independent experiments. B – C. SEL1L antibody does not recognize TPD52. B: To exclude possible TPD52 protein recognition by anti-SEL1L antibody, lysates (50 µg) obtained from 293FT cells transfected with myc-tagged TPD52 isoform 1 or empty vector (mock) were resolved by SDS-PAGE (10%) and probed with anti-myc, anti-SEL1L and anti-TPD52 antibodies recognizing TPD52 isoforms 1 and 2. Exogenous tagged TPD52 isoform 1 acquired a molecular weight similar to that of endogenous p38 (arrows for exogenous TPD52 isoform 1 and asterisks for endogenous p38), interfering with the evaluation of SEL1L antibody cross-reactivity. However, no increase of reactivity was observed in cells transfected with *myc-TPD52* isoform 1. Both myc and TPD52 antibodies selectively detected the exogenous protein (see arrows), confirming correct translation. C: Lysates (50 µg) from 293FT cells transfected with GFP-tagged TPD52 isoform 1 or empty vector (mock) were resolved by SDS-PAGE (10%) and probed with anti-GFP, anti-SEL1L and anti-TPD52 antibodies. Exogenous tagged TPD52 isoform 1 acquired a molecular weight of 58 KDa, well distinguishable from the endogenous SEL1L bands (see arrows for exogenous TPD52 isoform 1 and asterisks for endogenous p38 and SEL1LA). No or barely detectable reactivity with SEL1L antibody was observed in cells transfected with TPD52 isoform 1. Both GFP and TPD52 antibodies selectively detected the exogenous protein, confirming correct translation. D: TPD52 protein levels are unaffected by SEL1L small interfering RNA (siRNA): Lysates obtained from the same samples described in Figure 1 C were resolved by SDS-PAGE (12%) and blotted with anti-TPD52 antibody. While SEL1LA, p28 and p38 decreased close to 55%, 30% and 16% respectively, compared to cells treated with scrambled siRNA (see Figure 1C), TPD52 levels did not change. Vinculin was used as a loading control. E: TPD52 secretion is enhanced in SKBr3 cells exposed to DTT: Lysates and secreted proteins obtained from the samples described in Figure 2A were resolved by SDS-PAGE (12%) and blotted with anti-TPD52 and anti-vinculin antibodies. ER stress/UPR slightly promoted TPD52 secretion in the culture medium. The image is representative of two independent experiments.
(TIF)

Figure S6 **SEL1LB and -C transcripts are up-modulated in cancer cell lines.** RNAs extracted from KMS11, MCF10A and SKBr3 cells were analyzed by RT-PCR using primers specific for *SEL1LB* and *-C*. Signals shown here were obtained with 30 cycles for both isoforms. *HPRT* was used as a loading control. The *SEL1LB* and *–C* transcripts were up-modulated in the tested tumor cell lines relative to the non-tumorigenic MCF10A line. The image is representative of three different assays based on independent experiments.
(TIF)
