# Supplementary figures and images for: Secretion of Novel SEL1L Endogenous Variants Is Promoted by ER Stress/UPR via Endosomes and Shed Vesicles in Human Cancer Cells
Source: PLoS One. 2011 Feb 17;6(2):e17206. doi: 10.1371/journal.pone.0017206 (PMC3040770; doi:10.1371/journal.pone.0017206)

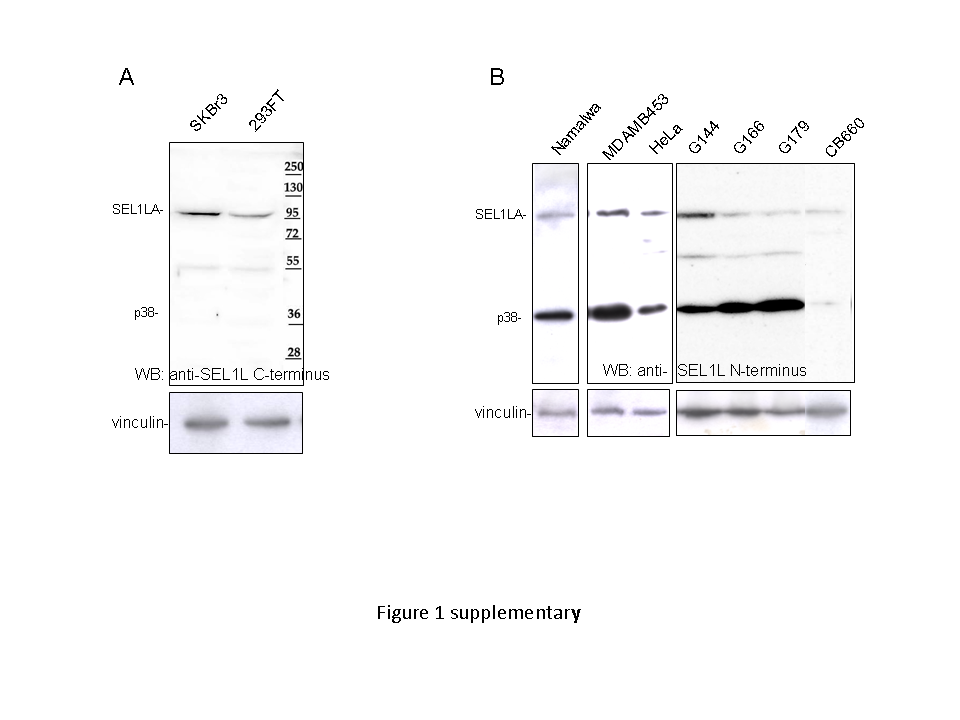

Supplement: Figure S1 — P38 and p28 are not identified by antibody against the SEL1L C-terminus and are detected in cancer cells of various origin by antibody against the SEL1L N-terminus. A. P38 and p28 are not recognized by polyclonal antibody against the SEL1L C-terminus: Lysates (50 µg) from 293FT (embryo kidney) and SKBr3 (breast cancer) cells were resolved by SDS-PAGE (10%) and probed with polyclonal anti-SEL1L C-terminus. Vinculin was used as a loading control. The polyclonal C-terminal SEL1L antibody recognized the ER-resident SEL1LA protein (95 KDa), but not the p38 and p28 forms. The blot is representative of three independent experiments. B. p38, detected with monoclonal antibody against the SEL1L N-terminus, is more evident in cancer cells of various origins relative to a normal human fetal brain cell line: Lysates (50 µg) from Namalwa (lymphoma), MDAMB453 (breast cancer), HeLa (cervical cancer), G144, G166 and G179 (glioblastoma) and CB660 (human fetal brain) cells were resolved by SDS-PAGE (10%) and probed with monoclonal anti-SEL1L N-terminus. Vinculin was used as a loading control. P38 was expressed at much higher levels in the tested cancer cell lines relative to CB660, while p28 was undetectable. A higher band of approximately 60 KDa may represent an additional SEL1L-related form expressed in glioblastoma cell lines. (TIF) [file pone.0017206.s001.tif]

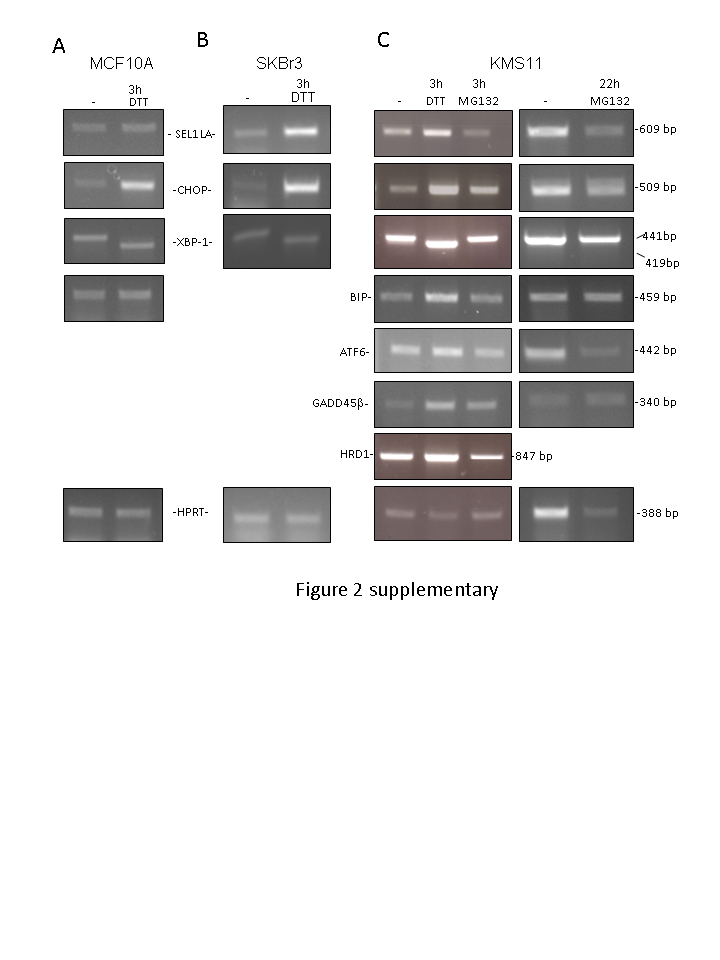

Supplement: Figure S2 — UPR studies in MCF10A and SKBr3 cells treated with DTT and in KMS11 cells treated with DTT and MG132. A. RT-PCR analysis of DTT-treated MCF10A cells: UPR activation was analyzed by RT-PCR in the samples described in Figure 2A1. UPR activation was confirmed by XBP-1 splicing and up-modulation of BIP and CHOP. HPRT serves as internal control. The image is representative of two different assays based on independent treatments. B. RT-PCR analysis of DTT-treated SKBr3 cells: RNA was extracted from the samples described in Figure 2B and analyzed by RT-PCR for the UPR. UPR activation was confirmed by XBP-1 splicing and CHOP up-modulation, concomitantly with increase of SEL1LA. HPRT serves as internal control. The image is representative of five different assays based on independent treatments. C. RT-PCR analysis of DTT- and MG132-treated KMS11 cells: UPR activation was assessed by RT-PCR on KMS11 cells treated with DTT or with MG132. UPR activation upon DTT treatment was confirmed by XBP-1 splicing and CHOP, BIP and ATF6 up-modulation; concomitantly SEL1LA also increased. MG132 treatment for 3 hrs resulted in an increase of CHOP and GADD45β, but there was no evidence of XBP-1 splicing and BIP and ATF6 modulation. Concomitantly, SEL1LA and HRD1 decreased. After 22 hours of MG132 treatment, BIP, CHOP and GADD45β increased. HPRT serves as internal control. The image is representative of five different assays based on independent treatments. (TIF) [file pone.0017206.s002.tif]

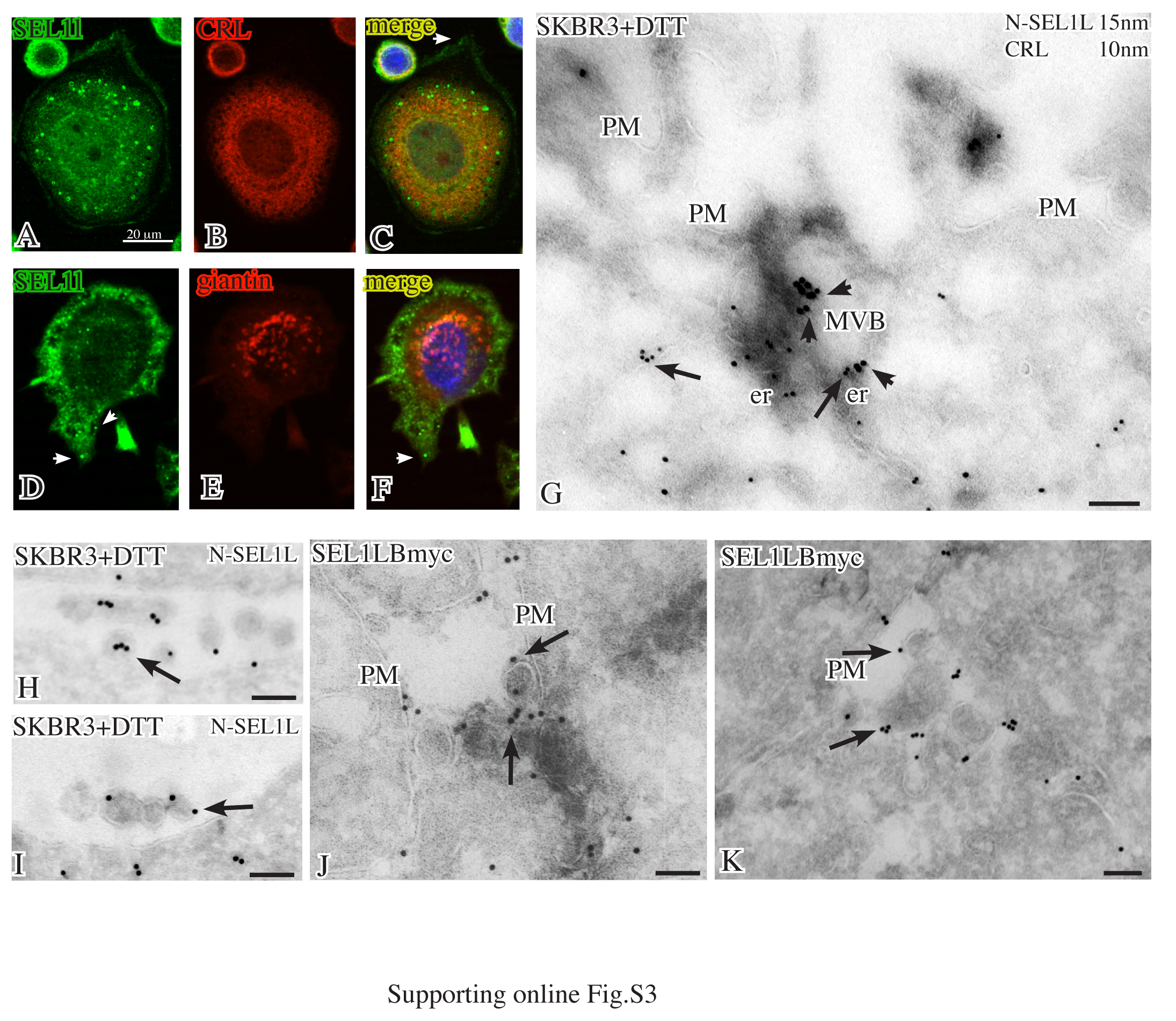

Supplement: Figure S3 — Localizations of SEL1L in DTT-treated SKBr3 cells and of myc-tagged exogenous SEL1LB in transfected 293FT cells. Immunofluorescence shows that in DTT-treated SKBr3 cells N-terminal SEL1L (green) intensely labels peripheral areas negative for the endoplasmic reticulum marker calreticulin and for the Golgi marker giantin (panels A–F). Cryoimmunogold electron microscopy of DTT-treated SKBr3 cells shows N-terminal SEL1L labeling in multivesicular bodies (panel G, arrowhead), on endoplasmic reticulum profiles, identified by calreticulin (panel G arrows), and in vesicles released from the plasma membrane after fission of the stalk (panels H-I, arrows point to stalks). Similarly, in SEL1L-Bmyc-transfected 293FT cells, exogenous myc-tagged SEL1LB labeling was detected along plasma membranes and in vesicles emerging from plasma membrane (panels J–K, arrows). Bars: 0.1 µm; er: endoplasmic reticulum; MVB: multivesicular body; PM: plasma membrane. (TIF) [file pone.0017206.s003.tif]

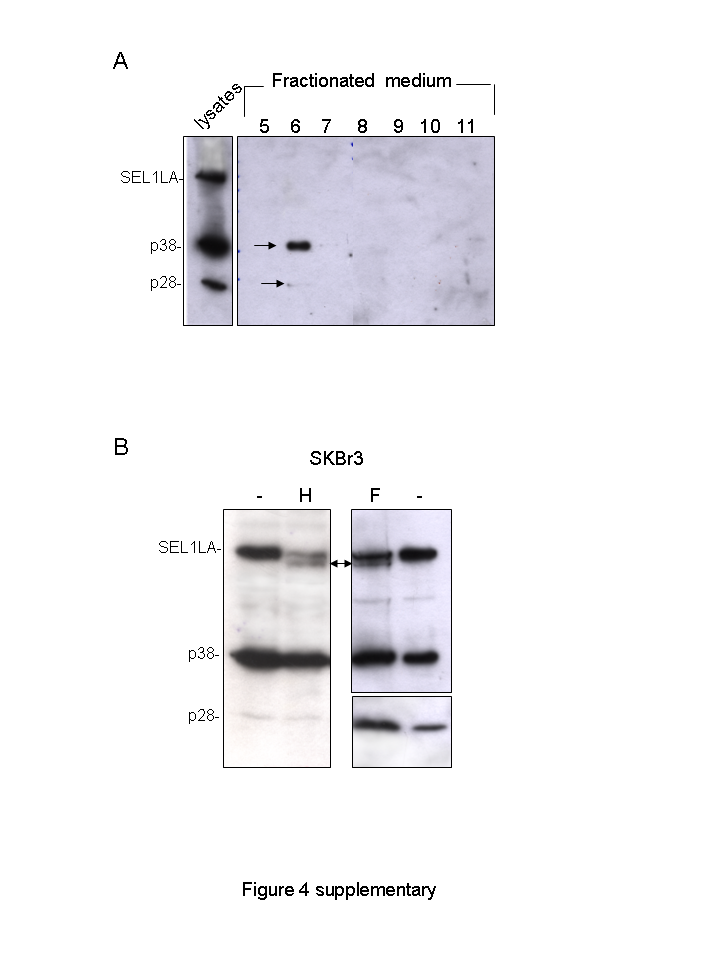

Supplement: Figure S4 — Biochemical characterization of SEL1L variants. A. Off-gel electrophoresis and Western blot analysis: Off-gel electrophoresis coupled with Western blot analysis was used to analyze the pIs of p38 and p28. The proteins extracted from the medium of DTT-treated SKBr3 cells were fractionated according to their pI using an Off-gel 3100 fractionator (Agilent Technologies) and aliquots of these fractions were analyzed by Western blot with monoclonal anti-SEL1L antibody. Both p38 and p28 (arrows) were detected in the sixth fraction, corresponding to the pI range of 5.25–5.50. B. N-glycosidase F (PGNase F) and endoglycosidase H (Endo H) digestions: SKBr3 cell lysates (80 µg) were incubated with endo H (H) and PGNase F (F), fractionated on SDS-PAGE (10%) and blotted with monoclonal anti-SEL1L antibody. Both p38 and p28 are PNGase F and endo (H) resistant. Note the mobility shifts of treated SEL1LA (see arrow). (TIF) [file pone.0017206.s004.tif]

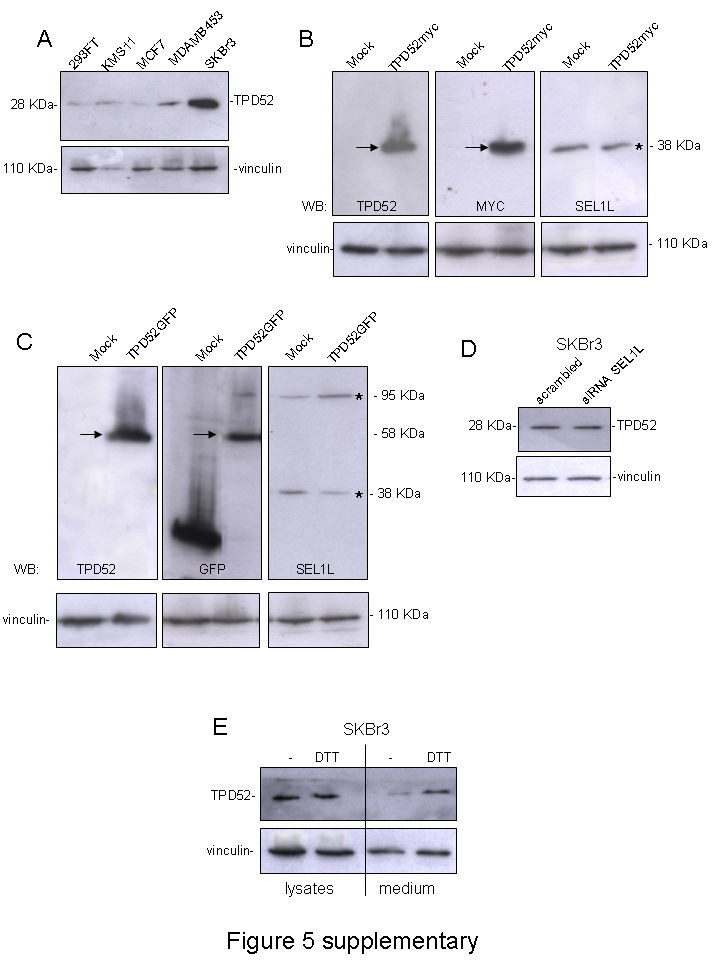

Supplement: Figure S5 — TPD52 analysis. A. TPD52 protein expression: Lysates (50 µg) from different cell lines, including 293FT (embryonic kidney), KMS11 (multiple myeloma), MCF7, MDAMB453, and SKBr3 (breast cancer) were resolved by SDS-PAGE (10%) and probed with polyclonal anti-TPD52 antibody. Vinculin was used as a loading control. SKBr3 cells over-expressed TPD52. The blot is representative of three independent experiments. B – C. SEL1L antibody does not recognize TPD52. B: To exclude possible TPD52 protein recognition by anti-SEL1L antibody, lysates (50 µg) obtained from 293FT cells transfected with myc-tagged TPD52 isoform 1 or empty vector (mock) were resolved by SDS-PAGE (10%) and probed with anti-myc, anti-SEL1L and anti-TPD52 antibodies recognizing TPD52 isoforms 1 and 2. Exogenous tagged TPD52 isoform 1 acquired a molecular weight similar to that of endogenous p38 (arrows for exogenous TPD52 isoform 1 and asterisks for endogenous p38), interfering with the evaluation of SEL1L antibody cross-reactivity. However, no increase of reactivity was observed in cells transfected with myc-TPD52 isoform 1. Both myc and TPD52 antibodies selectively detected the exogenous protein (see arrows), confirming correct translation. C: Lysates (50 µg) from 293FT cells transfected with GFP-tagged TPD52 isoform 1 or empty vector (mock) were resolved by SDS-PAGE (10%) and probed with anti-GFP, anti-SEL1L and anti-TPD52 antibodies. Exogenous tagged TPD52 isoform 1 acquired a molecular weight of 58 KDa, well distinguishable from the endogenous SEL1L bands (see arrows for exogenous TPD52 isoform 1 and asterisks for endogenous p38 and SEL1LA). No or barely detectable reactivity with SEL1L antibody was observed in cells transfected with TPD52 isoform 1. Both GFP and TPD52 antibodies selectively detected the exogenous protein, confirming correct translation. D: TPD52 protein levels are unaffected by SEL1L small interfering RNA (siRNA): Lysates obtained from the same samples described in Figure 1 C were resolv [file pone.0017206.s005.tif]

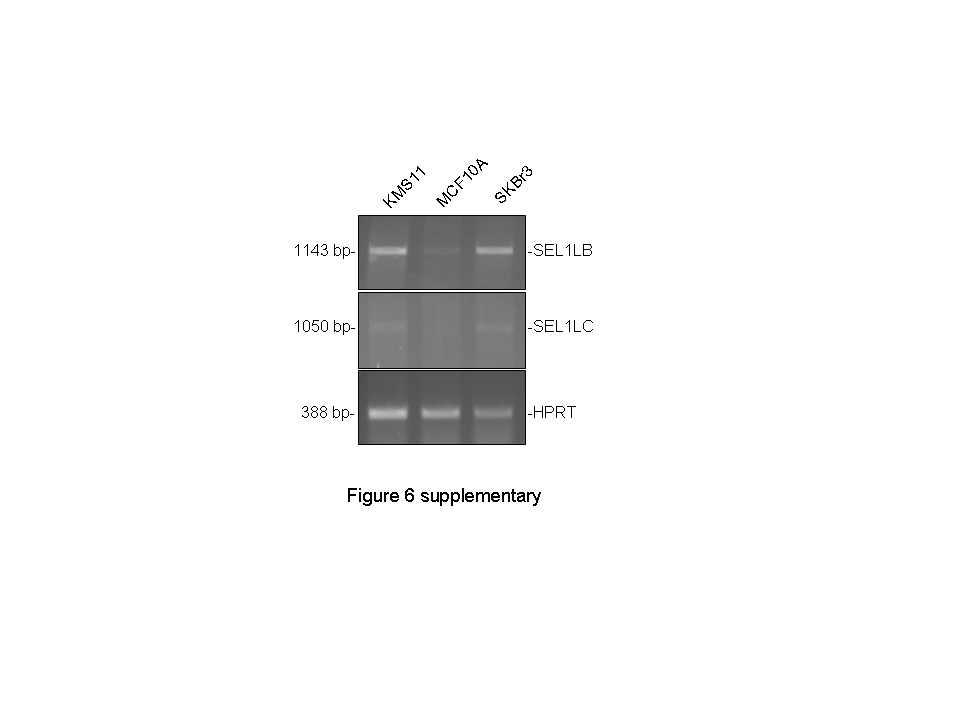

Supplement: Figure S6 — SEL1LB and -C transcripts are up-modulated in cancer cell lines. RNAs extracted from KMS11, MCF10A and SKBr3 cells were analyzed by RT-PCR using primers specific for SEL1LB and -C. Signals shown here were obtained with 30 cycles for both isoforms. HPRT was used as a loading control. The SEL1LB and –C transcripts were up-modulated in the tested tumor cell lines relative to the non-tumorigenic MCF10A line. The image is representative of three different assays based on independent experiments. (TIF) [file pone.0017206.s006.tif]
